# Improving the performance of air conditioning unit by using a hybrid technique

**DOI:** 10.1016/j.mex.2022.101620

**Published:** 2022-01-15

**Authors:** Kayser Aziz Ameen, Husam Abdulrasool hasan, Mustafa J. Al-Dulaimi, Azher M. Abed, Haidar F. Al-Qrimli

**Affiliations:** a Department of Air conditioning and Refrigeration, Al-Esraa University College, Baghdad, Iraq; b Department of Air conditioning and Refrigeration, AL-Mustaqbal University College, Babylon, Iraq; c Petroleum Contracts & Licensing Directorate Ministry of Oil, Baghdad, Iraq

**Keywords:** The performance of Air Conditioning Unit, HVAC, Air refrigeration system, Finned tubes

## Abstract

An Air Conditioning Unit with magnetic field and different tubes was designed, fabricated and evaluated in this study. The Effect of magnetic field and different types of tubes on the performance of Air Conditioning was studied experimentally. A testing system of Air Conditioning Unit was developed as the test rig. The modified tubes as a straight tube before the condenser and after the evaporator were replaced by a finned bended tube with five bends and a coil finned tube with five turns. The experimental results for the temperature of refrigerant and the coefficient of performance for an air conditioning unit were presented. Changing the tubes and introducing electric charging has a significant effect on the performance of the unit. The electric charging has a positive effect of the performance of the system. The electric charging enhanced the performance by 76% in case of bent tube and by 177% in case of coil tube. The bent pipe increases the refrigerant temperature between 50% and 200%, while the coil pipe increases the temperature between 18 % and 190 %.

• This method increases the refrigerant temperature for Air Conditioning system.

• This method provides simple technical testing of Air Conditioning Unit with magnetic field and different tubes

• This method can be useful to enhance the performance of Air Conditioning Unit.

## Graphical abstract

**Figure:**
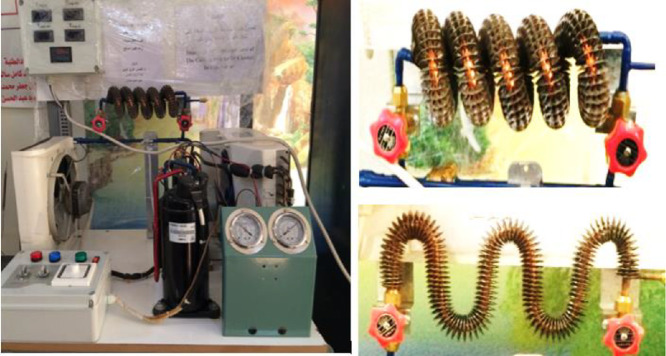

Specification table

**Table:** 

Subject Area:	Thermal system
More specific subject area:	Air Conditioning
Method name:>	Designed, fabricated and tested Air Conditioning system
Name and reference of original method:	Improving the performance of Air Conditioning Unit by Using a Hybrid Technique
Resource availability:	The data are available in this article.

## Introduction

Due to the increasing demand of electric power, the raising of performance of air conditioning devices should be without increasing their power consumption. Many passive techniques were used to improve the performance of air conditioning devices. These techniques included of using alternative refrigerants, increasing surface area of condensers, changing pipes diameter and pipes configuration. [1] performed an experimental investigation to choose alternative refrigerant for a domestic refrigerator. The tested refrigerant are R-12, R-134a, and Hydrocarbonic mixture (R-290, R-600a) of mass ratio (61%,39%) and Hydrocarbonic mixture (R-134a , R-22) of mass ratio (52% , 48%).The experimental and theoretical, results are in a good agreement. It was found that the use of R-134a results in higher power consumption by (7-5%) as compared with R-12. (R-290, R-600) mixture was found to has a lower energy consumption by 4% than R-12 when using mineral oil. Also the use of (R-134a, R-22) mixture leads to a decrease in the power consumption by 12% as compared with R-12. [2] investigated experimentally the performance of air refrigeration system by using two refrigerant R-12 and R-134a especially in the condenser. The results showed that the coefficient of performance of R-2 is higher that of R-134a at the same conditions it was found that the heat rejected by condenser is decreased by increasing ambient temperature.

[3] Conducted an exergy analysis based on experimental results to study the effect of replacement of R-12 refrigerate by R-413A on the performance of a domestic refrigeration system designed to work on R-12. It was found that the power consumption in the compressor is always lower for R-413A than for R-12. Also the exergy efficiency for the system with R-413A is better than for that working with R-12. It was indicated to the possibility of replacing R-12 with R-413A without the need to replace or modify any part of the cycle. [4] Studied experimentally the effect of varying capillary tube diameter of length (130 cm) on the performance of a refrigeration compression system using (R-134a). also they studied theoretically the performance of the system using refrigerants (R-134a, R-12, R-500 and R-152) to examine the effect of capillary tube varying. The results for refrigerants (R134a, R152) showed that the coefficient of performance decreased by (11.3%, 22.8%) respectively with the increase of mass flow rate (58.7%). Guobing and Zang [5] have conducted an experimental investigation on helically coiled capillary tube with R22 refrigerant. The mass flow rate of coiled capillary tubes was found to by (6 – 15%) than that of straight tube. Also it was found it the coil diameter reduced from 0.3m to 0.04m, the capillary tube length is reduced by about 10% which improves of the performance of the system.The demand of minimizing the high cost and energy resulted in many efforts to enhance the performance of cooling systems [6]. The techniques of heat transfer enhancement have been classified into two categories such as passive and active techniques [7], [8], [9], [10], [11], [12], [13], [14], [15], [16], [17], [18], [19], [20], [21], [22]. The massive increase in population across the world has caused a very high demand on resources including water, electricity, and housing. These demands have caused a large increase in the consumption of fossil fuels. Many technique was used to enhance the efficiency of air conditioning system.one of these techniques is Passive which include the modification of the heat transfer surface by attaching fins, baffles, tabulators and changing the shape of the surface. Passive techniques require no power. The active techniques require external power to augment the heat transfer. such these techniques are applying vibrational motion or magnetic field. In the present work a hybrid of the two techniques is used to enhance the performance of an air conditioning unit. Some parts of the tubes are replaced by fined bent and finned coil tube with applying of electric charging. The studies on the increase of convection heat transfer by electric fields can support to tackle the problem in a reliable and stable manner. One of the passive reinforcement techniques is electric field augmented convective heat transfer. Previously, researchers concentrated on the electrohydrodynamic impacts on heat transfer enhancement [23,24]. The experimental results shown that the intensity of the applied electric field has a significant impact on the heat transfer characteristic. As the electric field strength increasing, so was the heat transfer enhancing effects

[25], [26], [27]. The results demonstrated that in the absence of an electric field, bubble detachment did not occur at low gas flow rates; nevertheless, at higher gas flow rates, the dynamical effects were sufficient to induce bubble detachment even in the lack of buoyancy. The use of an electric field was shown to be useful in inducing bubble separation at diameters bigger but of the same order of magnitude as under normal gravity, as well as in generating a force to pull the bubbles away from the orifice. [28], [29], [30] The electric field forces remodel the natural convection-generated velocity and temperature fields. The local electric field improves the local thermal conductivities of nanofluid. A dimensionless quantity dubbed the electric convection number, as well as fitting equations, are developed and investigated to reveal the connection between natural convection and the EHD effect. Furthermore, under an electric field, the motions of the nanoparticle and the basefluid are inverse, which is helpful to the stability of the nanoparticle in the basefluid [31], [32], [33], [34] [35,36].

The objective of the present study was to identify the effect of electric charging and tubes geometry in order to improve the performance of air conditioning unit.

## Experimental setup

**Fig. 1:**
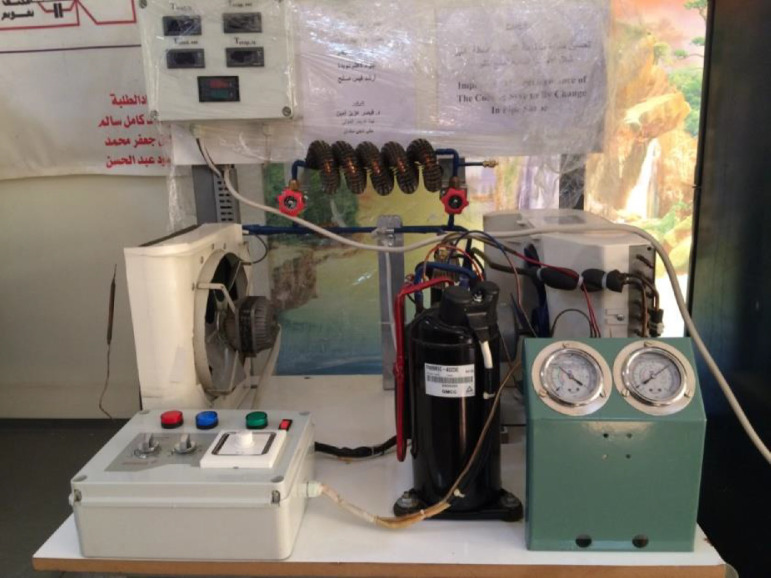
The test rig.

The test rig used for the experiments is show in Fig. 1. The refrigerant is R140A. It consists mainly from a compressor, a condenser, a capillary tube and an evaporator.

**The compressor**: is 0.75 hp rotary type and was selected based on the capacity of the evaporator.

**The condenser**: is air cooled with fan and consists of two rows and nine columns of copper tube of diameter (4mm).

**The evaporator**: is of finned tubes and consists of two rows and eight columns of copper tube of diameter (4 mm).

**The capillary tube**: is made of copper and of length (109 mm) and diameter (1 mm).

**Fig. 2:**
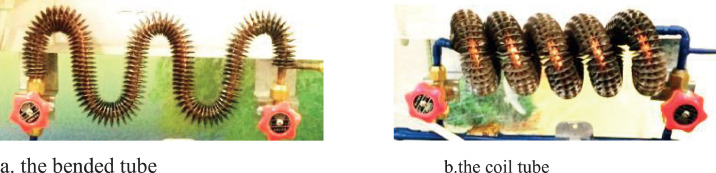
Pipe configurations. a. the bended tube. b.the coil tube.

**Fig. 3:**
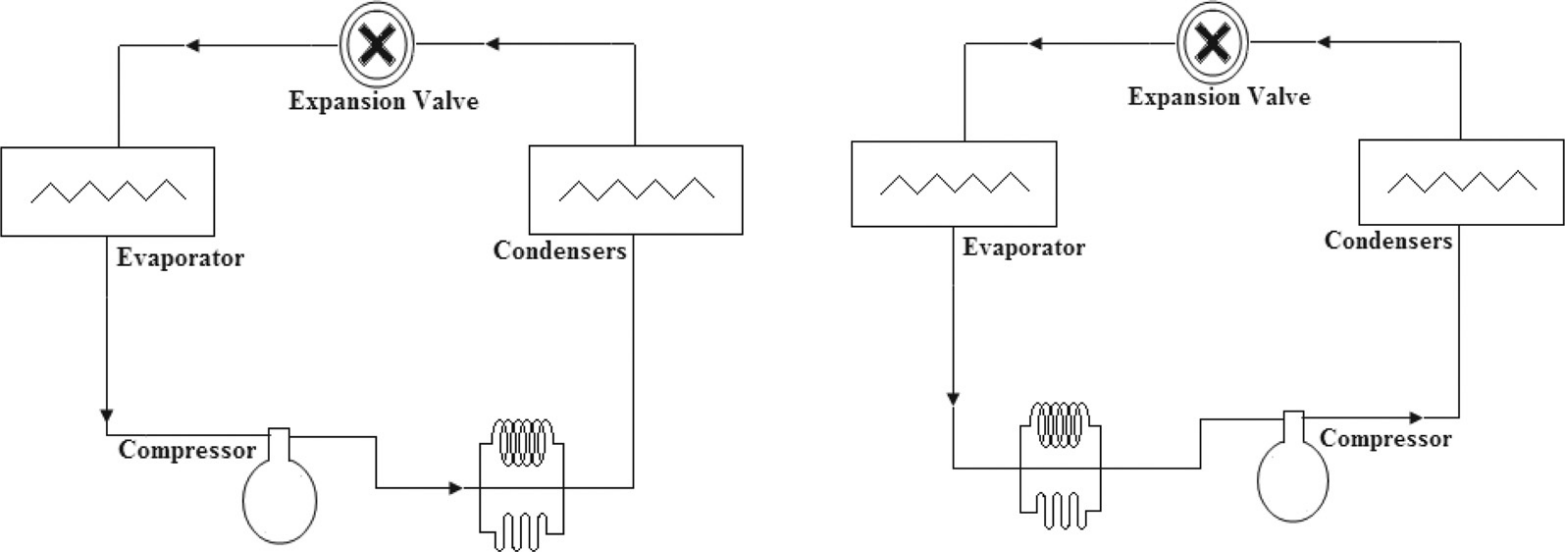
Modified tube location.

**The modified tube**: the straight tubes before the condenser and after the evaporator were replaced by a finned bended tube with five bends and a coil finned tube with five turns as shown in Figs. 2 and 3

### Electric Charging

A 100 volt AC source is connected to the tubes of the refrigerant at point before the bended and coil tube in order to investigate the effect of electric current on the performance of the window type air conditioner.

## Methodology

The experiments were conducted under constant room conditions. The experiment conducted first for straight tube after the condenser and before the evaporator then the straight tube is replaced by a bended tube and a coil tube. After replacing the tube, the electric charge is applied at a point before the bended tube and coil tube.

## Experimental error and uncertainty analysis

Determination uncertainty in the measured results of experimentation is important. The experimental uncertainties were calculated by applying Gauss propagation law. The result R is to be calculated as a function of the independent variables x_1_, x_2_, x_3_, …, x_n_ and w_1_, w_2_, w_3_, …, w_n_ represents the uncertainties in the independent variables. Then, uncertainty R is expressed as:

$$W_{R}={\left[{\left(\frac{\partial R}{\partial x_{1}}w_{1}\right)}^{2}+{\left(\frac{\partial R}{\partial x_{2}}w_{2}\right)}^{2}+\ldots +{\left(\frac{\partial R}{\partial x_{n}}w_{n}\right)}^{2}\right]}^{1/2}$$

The independent parameters measured in the experiments: voltage, current, pressure, ( inlet, outlet, and ambient) temperatures were carried out by to measure the experiments uncertainties. The experimental uncertainties associated were shown in Table 1.

**Table 1:** Uncertainties associated with the individual elements of the Air Conditioning system.

Equipment	Parameter	Experimental uncertainty
pressure gauges	pressure	± 3.2 %
Thermocouples	Temperature (inlet, outlet, and ambient)	± 1.09°C
Multimeter	Voltage	± 0.05 %
Multimeter	Current	± 0.003 %

### Experimental errors

**Fig. 4:**
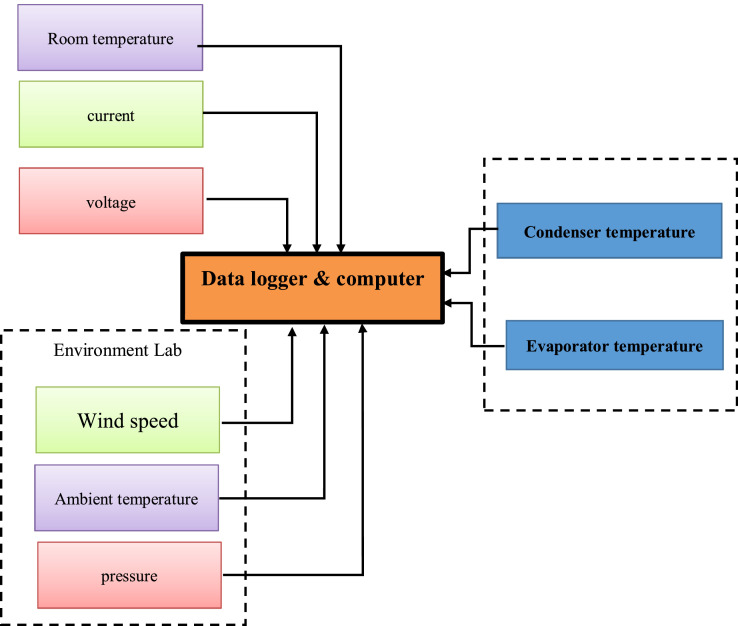
The measuring system for AC unit.

In the research, error for the performance had been derived and calculated. Error for pressure is around ± 3.2 %while error for Temperature is around ± 1.09°C as shown in Fig. 4. The error occurred depend on the instrument of measurement. Error for Temperature was lower than pressure. Errors for Multimeter are around ± 0.05 %,± 0.003 % for voltage and current, respectively.

### testing systems

The fabricated Air Conditioning system with different paramters need to undergo certain testing at lab. The lab (indoor) testing is important because in the lab only, room temprtuer can be control, and accurate measurement can be obtained. Although the lab (indoor) testing is important, the space testing need to be conducted enable to investigate the performance of the AC system to the real time situation. Due to that purpose, the setup equipment can be used for Lab testing. 0.75 hp compressor rotary type was selected based on the capacity of the evaporator. fan and two rows and nine columns of copper tube of (4mm) was utilize as condenser with air cooled. The finned tubes was used as evaporator and consists of two rows and eight columns of copper tube of diameter (4 mm). copper tube with length of (109 mm) and diameter of (1 mm) was used as capillary tube. instrument was used to measurement(room tempture,voltage,current, Environment Lab)then send all data to the pc in order to record the all results and calculte the performance of AC system as shown in Fig. 4.

## Results

### Effect of tubes configuration before condenser

Figs. 2 and 3 show the effect of the tube configuration before condenser on the temperature of the refrigerant entering the compressor with and without electric charging. the coil tube seems to have the strongest effect of the temperature of refrigerant to result higher temperatures than straight and bent tube. this can be attributed to the higher turbulence caused by this type of pipes. The electric charging has a significant effect on the refrigerant temperature. This leads to a decrease in the temperature of the refrigerant

**Fig. 5:**
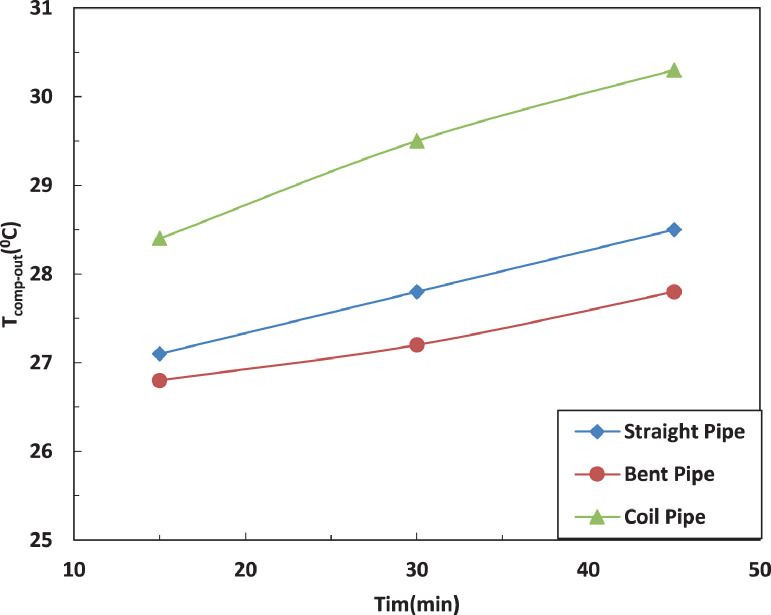
Effect of pipe configuration on refrigerant temperature entering the compressor.

**Fig. 6:**
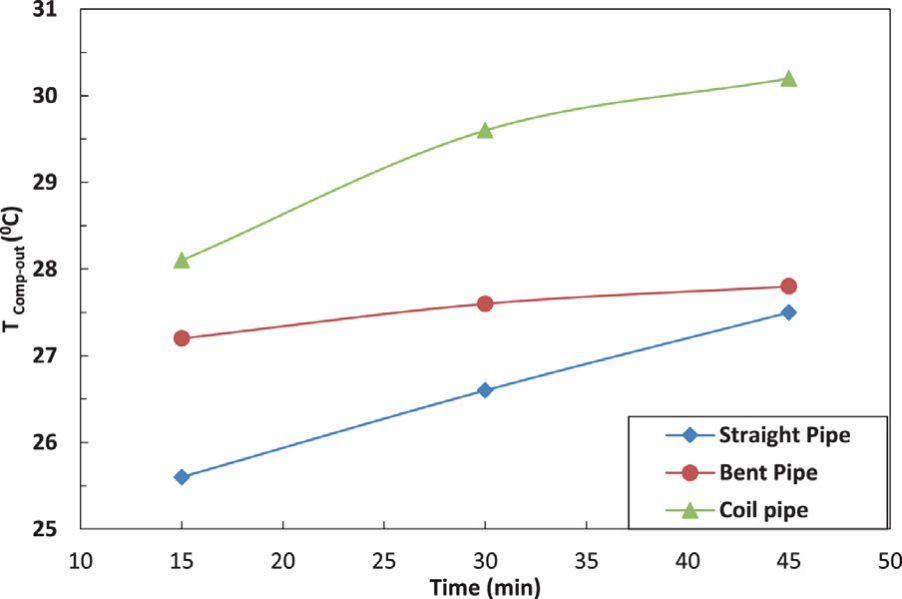
Effect of pipe configuration on refrigerant temperature entering the compressor with electric charging.

Figs. 5 and 6 show the effect of the tube configuration before condenser on the temperature of the refrigerant leaving the compressor with and without electric charging. The bent tube has the strongest effect of the temperature of refrigerant leaving the compressor to lead in higher temperatures than straight and coil tube. The electric charging has a significant effect on the refrigerant temperature. This leads to a decrease in the temperature of the refrigerant

**Fig. 7:**
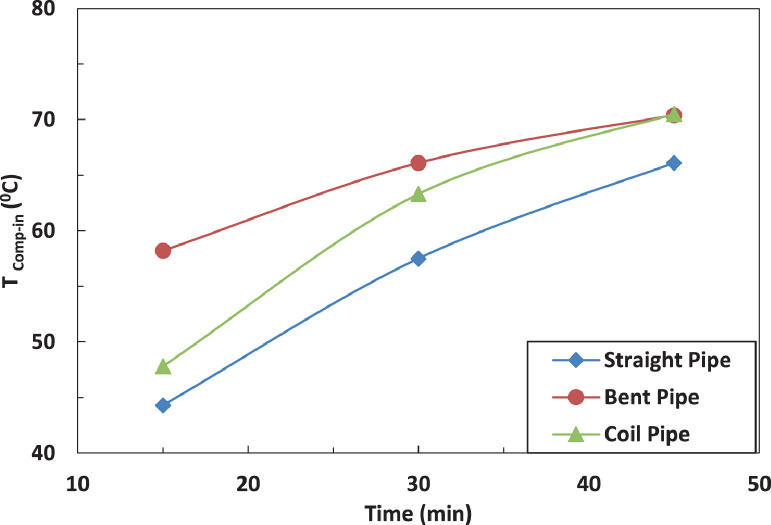
Effect of pipe configuration on refrigerant temperature leaving the compressor with electric charging.

**Fig. 8:**
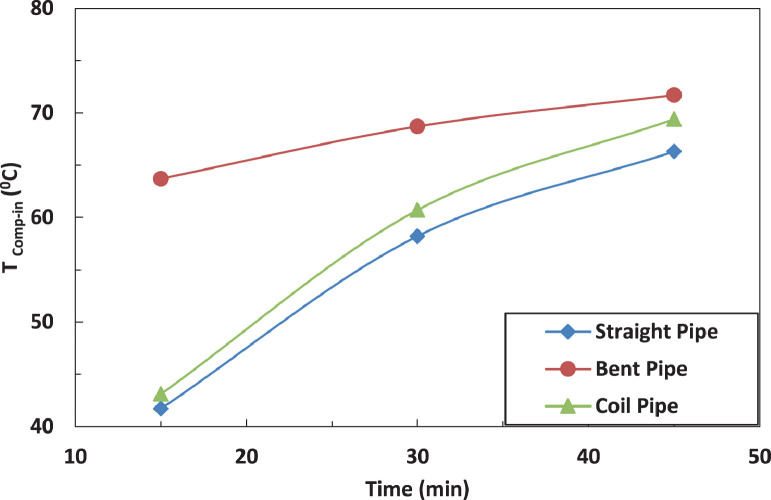
Effect of pipe configuration on refrigerant temperature leaving the compressor with electric charging.

The variation of temperature of refrigerant leaving the evaporator for different pipe configurations is shown in Figs. 7 and 8 without and with electric charging. The coil pipe seems to have the most significant impact on the temperature of refrigerant for both cases. The electric charging decreases the temperature of the refrigerant leaving the evaporator

**Fig. 9:**
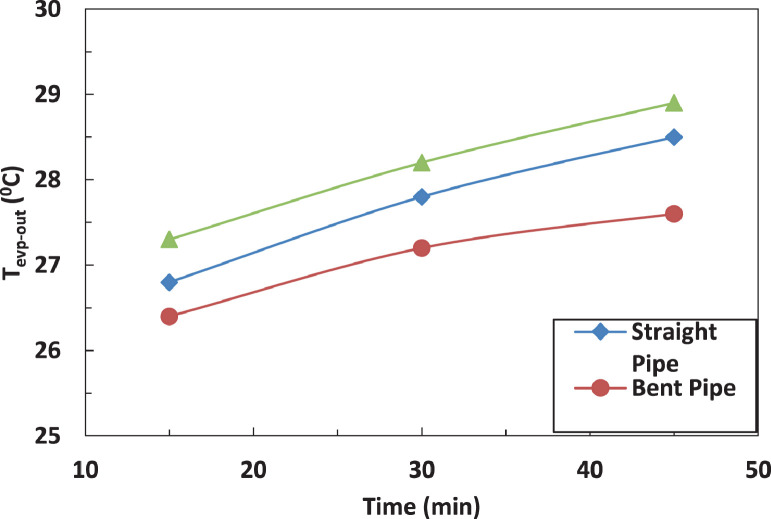
Effect of pipe configuration of temperature of refrigerant leaving the evaporator.

**Fig. 10:**
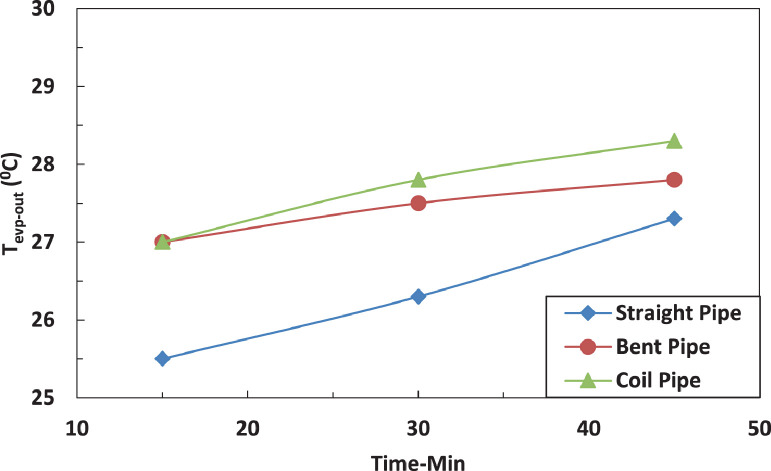
Effect of pipe configuration of temperature of refrigerant leaving the evaporator with electric charge.

The variation of temperature of refrigerant entering the evaporator for different pipe configurations is shown in Figs. 9 and 10 without and with electric charging. It seems that only the straight tube is significantly affected by the electric charging. The electric charging decreases the temperature of refrigerant for straight pipe.

**Fig. 11:**
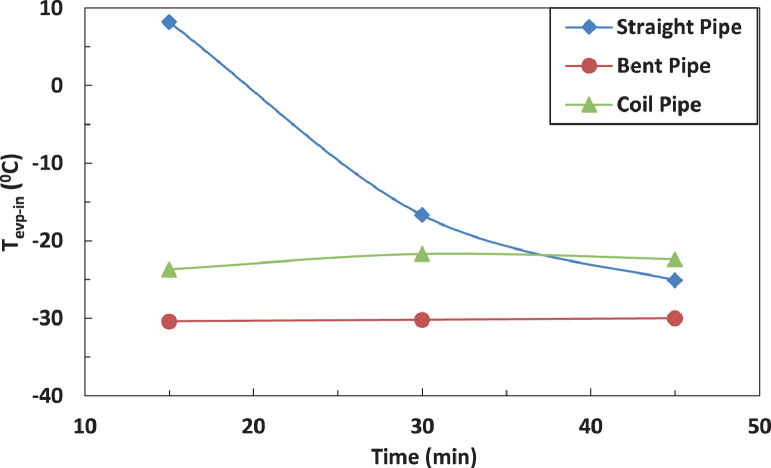
Effect of pipe configuration of temperature of refrigerant entering the evaporator without electric charge.

**Fig. 12:**
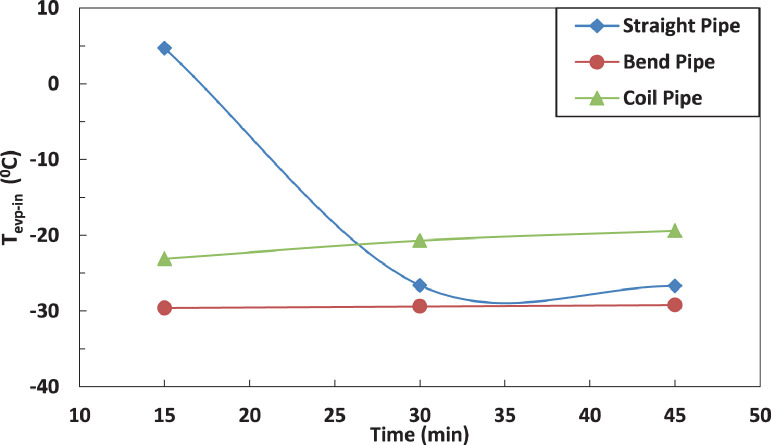
Effect of pipe configuration of temperature of refrigerant entering the evaporator with electric charge.

**Fig. 13:**
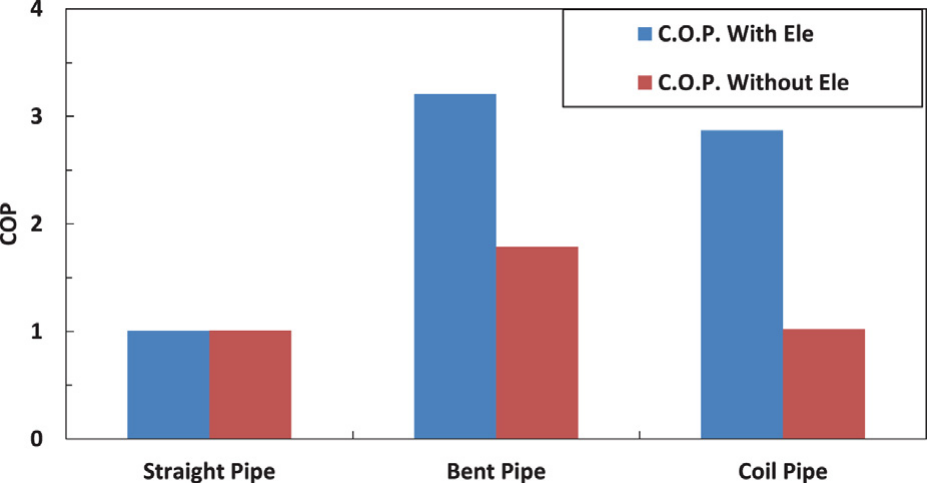
Effect of electric charging on COP for different pipe configuration before condenser.

Figs. 11,12 and 13 shows the effect of different pipe configurations of the coefficient of performance of the system. It can be noted that both bent and coil pipes enhanced the coefficient of performance but the bents pipe has the highest embankment. The electric charging has a positive effect of the performance of the system. The electric charging enhanced the performance by 79% in case of bent tube and by 181% in case of coil tube. Table 2 show the effect of electric filed on COP for different pipe configuration before condenser.

**Table 2:** effect of electric filed on COP for different pipe configuration before condenser.

Equipment	COP With Electric field	COP Without Electric field
Straight tube	1	1
Bent tube	3.2	1.7
Coil tube	2.87	1

### Effect of tubes configuration after the evaporator

**Fig. 14:**
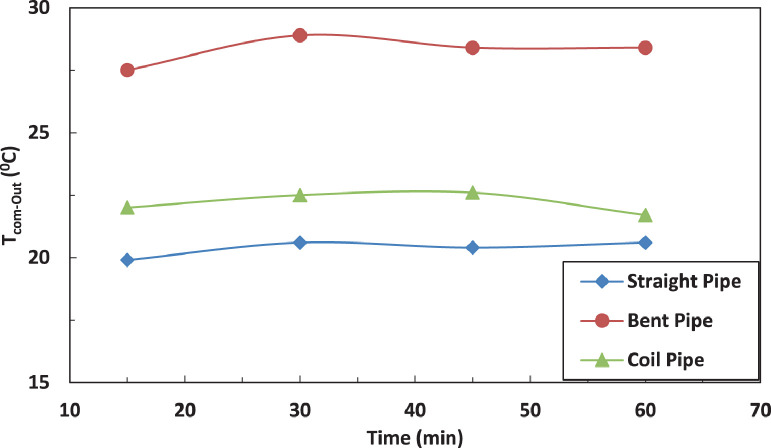
Effect of pipe configuration of temperature of refrigerant leaving the compressor without electric charge.

**Fig. 15:**
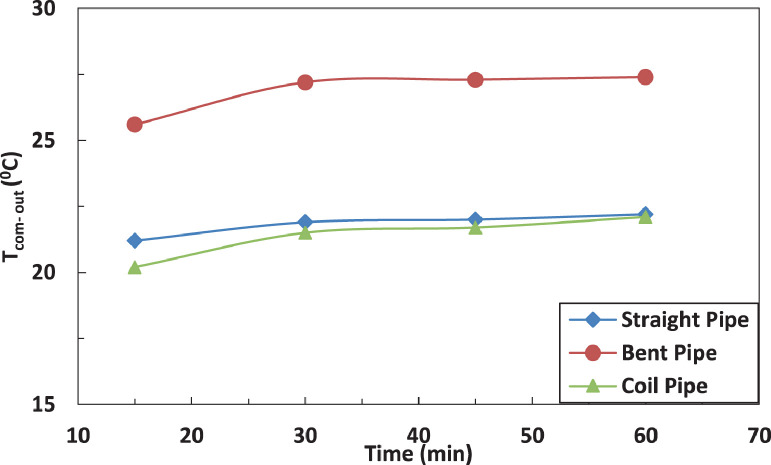
Effect of pipe configuration of temperature of refrigerant leaving the compressor with electric charge.

Figs. 14 and 15 show the effect of the tube configuration after the evaporator on the temperature of the refrigerant leaving the compressor with and without electric charging. It can be noticed that the pipe configurations have a significant impact on the refrigerant temperature. The coil tube increases the temperature by 10% as compared with straight tube. The bent tube has the maximum effect by increasing the temperature by 40% as compared with straight tube. The electric charging dampens the increase of the temperature of refrigerant to become 30% for bent tube and decreases the temperature by 1 % for coil tube.

**Fig. 16:**
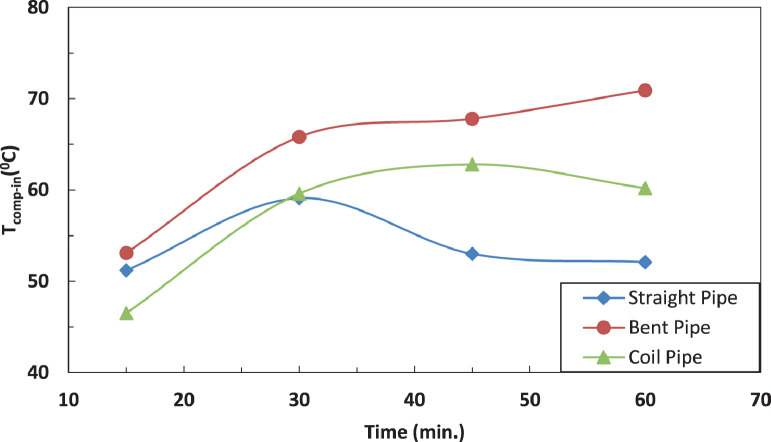
Effect of pipe configuration of temperature of refrigerant entering the compressor without electric charging.

Figs. 15 and 16 represent the effect of pipe configurations after evaporator on refrigerant temperature entering the compressor without and with electric charging. It seems that bent pipe increases the temperature by 4% initially and this ratio increases to reach 36 % after one hour. The coil tube initially decreases the temperature by 8% and then increases the temperature by 15 %. The electric charging tends to increase the temperature by nearly 40 % for bent tube and nearly 16 % for coil tube

**Fig. 17:**
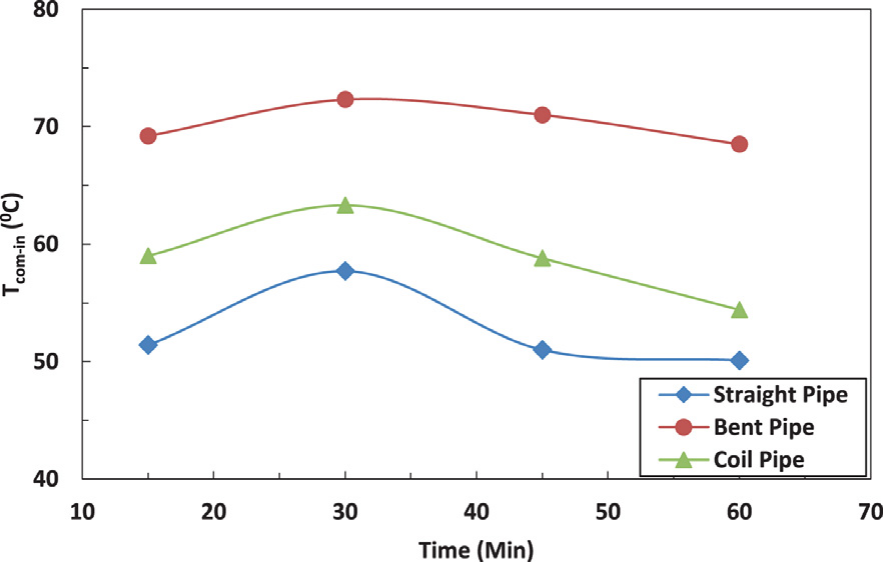
Effect of pipe configuration of temperature of refrigerant entering the compressor with electric charging.

**Fig. 18:**
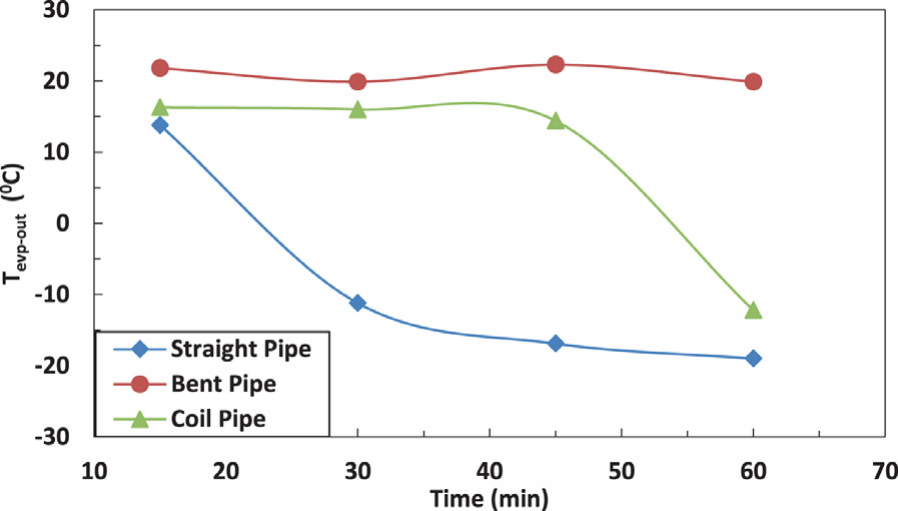
Effect of pipe configuration of temperature of refrigerant leaving the evaporator without electric charging.

The effect of tube configuration on temperature of refrigerant leaving the evaporator without and with electric charging is shown in Figs. 17 and 18. The bent pipe increases the refrigerant temperature between 50% and 200% , while the coil pipe increases the temperature between 18 % and 190 %. It ca be said that electric charging doesn't have a significant effect of the temperature of refrigerant for this case.

**Fig. 19:**
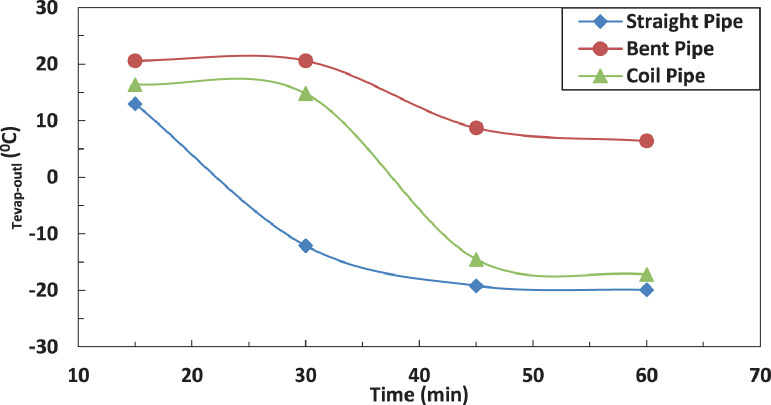
Effect of pipe configuration of temperature of refrigerant leaving the evaporator with electric charging.

**Fig. 20:**
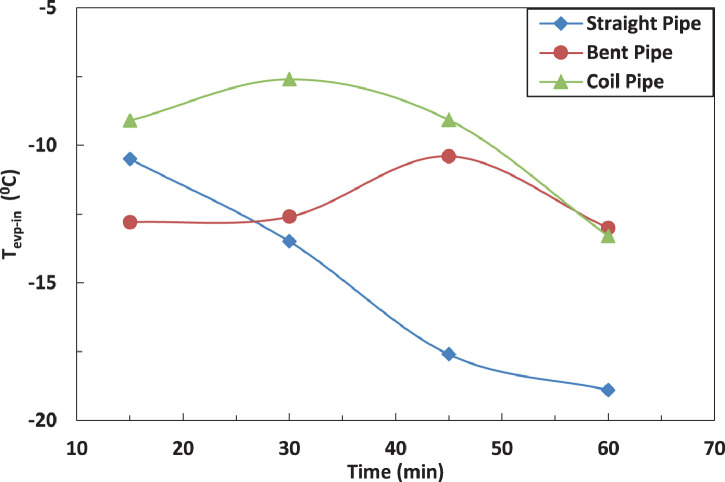
Effect of pipe configuration of temperature of refrigerant entering the evaporator without electric charging.

**Fig. 21:**
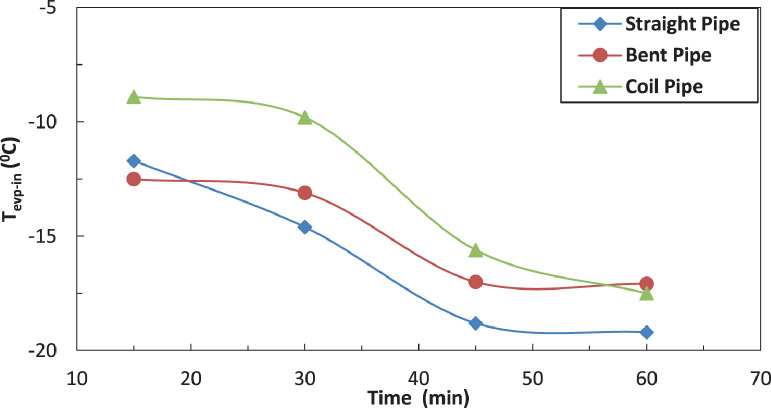
Effect of pipe configuration of temperature of refrigerant entering the evaporator with electric charging.

The effect of affection of the temperature of refrigerant entering the evaporator by pipe configurations without and with electric charging is shown in Fig. 19, 20 and 21. The coil pipe seems to have the strongest impact by increasing the temperature between 13 % and 30 % while the bent pipe initially decreases the temperature by 20 % then increases it by nearly 27 %. It can be noticed that the electric charging dampens the increase of temperature caused by pipe configurations.

**Fig. 22:**
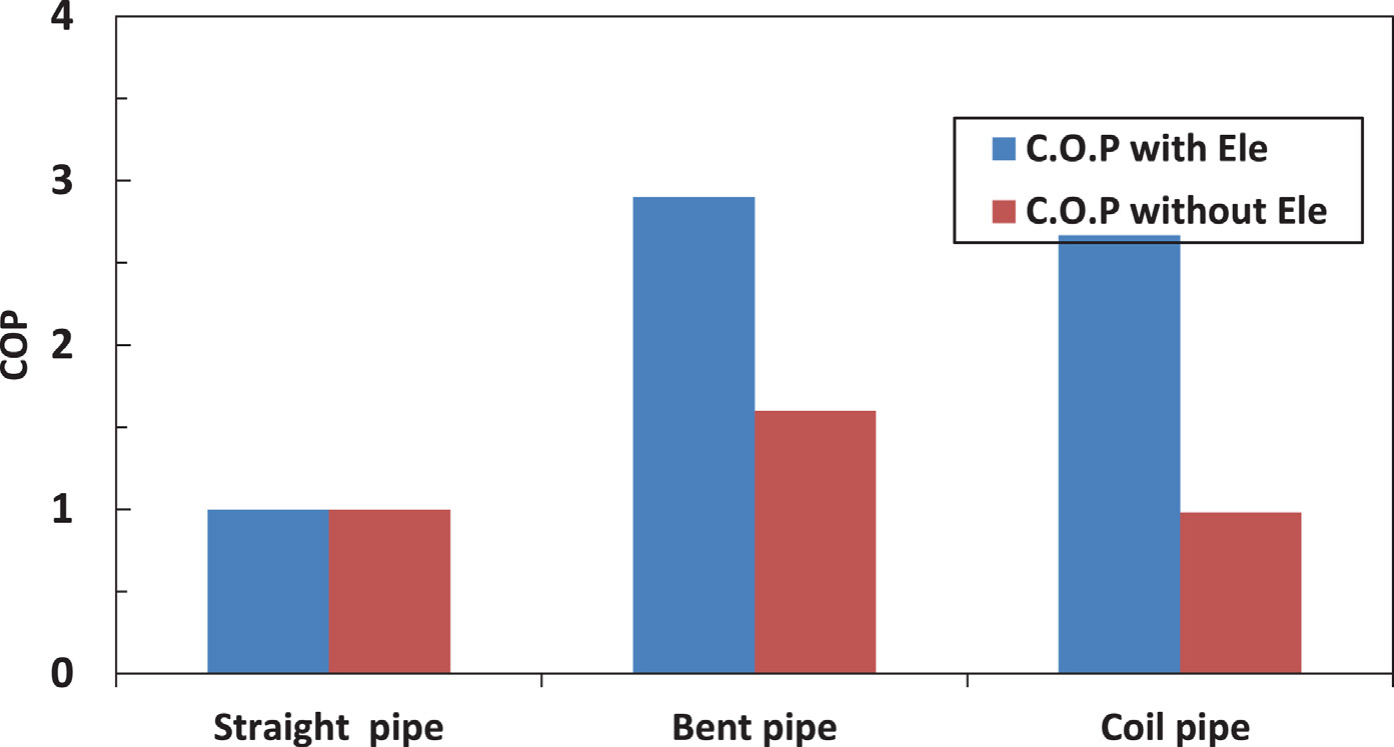
Effect of electric charging on COP for different pipe configuration.

Fig. 22 shows the effect of different pipe configurations after evaporator on the coefficient of performance of the system. It can be noted that both bent and coil pipes enhanced the coefficient of performance but the bents pipe has the highest enhancement. The electric charging has a positive effect of the performance of the system. The electric charging enhanced the performance by 76% in case of bent tube and by 177% in case of coil tube. Table 3 shows the effect of electric filed on COP for different pipe configuration after evaporator.

**Table 3:** effect of electric filed on COP for different pipe configuration after evaporator.

Equipment	COP With Electric field	COP Without Electric field
Straight tube	1	1
Bent tube	2.9	1.6
Coil tube	2.67	0.98

## Conclusions

The experimental results for the temperature of refrigerant and the coefficient of performance for an air conditioning unit are presented. Changing the tubes and introducing electric charging have a significant effect on the performance of the unit. The main conclusions from this study can be summarized as:

• Introducing the modified tube has a strong effect on the temperature of refrigerant and the performance of the unit.
• The bent tube has the biggest effect on the performance of the unit.
• Electric charging enhances the performance of the unit due to the increases kinetic of refrigerant particles

## Declaration of Competing Interest

None
